# The effect of combined resistance and home-based walking exercise in type 2 diabetes patients

**DOI:** 10.4103/0973-3930.57347

**Published:** 2009

**Authors:** Kucukarslan Aylin, Daskapan Arzu, Sayinalp Sabri, Tuzun Emine Handan, Alaca Ridvan

**Affiliations:** Trabzon Fizyotem Out Patient Clinic of Physical Therapy and Rehabilitation, Turkey; 1 Baskent University Faculty of Health Sciences, Department of Physical Therapy and Rehabilitation, Turkey; 2 Ankara Bayindir Hospital, Turkey

**Keywords:** Home-based walking, resistance training, type-2 diabetes

## Abstract

**Aims:**

The aim of this study was to evaluate the effect of combined resistance and home-based walking exercise on glycemic and metabolic control, depression and quality of life in type-2 diabetes patients.

**Methods:**

This prospective study was conducted at a private hospital in Turkey. Thirty-six type-2 diabetic patients participated in the study. Subjects were randomly distributed in one 8 week exercise intervention or in one control group. Exercise program consisted of resistance training and home-based walking. Before and after the training program, muscular strength, fasting blood glucose, hemoglobin A1C, (HbA1C) and plasma lipid values, quality of life and symptoms of depression of the patients were assessed.

**Results:**

Exercise group's baseline HbA1C was significantly higher than the control groups (p< 0.05); other blood parameters were similar between the two groups (p>0.05). At the baseline no significant differences were observed in the depression and four subscales (physical function, physical role, bodily pain, and general health perceptions) of the SF-36 between the exercise and control groups (p>0.05). The exercise group had higher scores of emotional role, vitality and mental health subscales than the control groups after the training programs (p<0.05).

**Conclusion:**

Exercise training which included resistance training and home- based walking could be safe, effective and beneficial in diabetic patients.

## Introduction

World Health Organization estimated that the numbers of people with diabetes will be more than double by 2030.[1] Main treatment goals of the type-2 diabetes are maintenance of good metabolic control, prevention of complications and improvement of psychological health and quality of life (QOL).[2,3]

It is well established that exercise can improve glycemic control, overall health and quality of life.[4–6] According to the American College of Sports Medicine, physical activity programs in patients with type-2 diabetes should include endurance and resistance training for developing and maintaining cardio respiratory fitness, body composition, and muscular strength and endurance.[7] Despite these benefits, literature stated that many of patients with diabetes have stopped the exercise programs.[8,9] Recently, it has been thought that, instead of structured aerobic exercise, unstructured, lifestyle physical activity is a good alternative for sedentary population and diabetic patients.[10–12]

The aim of this study was to evaluate the effect of combined resistance and home-based walking exercise on glycemic and metabolic control, depression and quality of life in type-2 diabetes patients.

## Materials and Methods

The study was approved by the Ethics Committee of the Bayindir Hospital. Thirty-eight type-2 diabetic patients (27 men and 11 women aged 54.3 ± 8 years) volunteered for the study. All participants gave written informed consent. Subjects were included if they were not currently and had not attended resistance training or aerobic exercise for the previous six months. Subjects were defined as having diabetes according to the criteria in the report of the expert committee on the diagnosis and classification of diabetes mellitus by the endocrinologist.[13] Exclusion criteria were as follows: older than 65 years, chronic alcohol use, smoking, history of coronary artery disease, renal impairment, hepatic impairment, hyperuricemia, uncontrolled hypertension (systolic blood pressure > 160 mm Hg, diabetic neuropathy or retinopathy, taking medications known to influence metabolism (insulin, diuretics, cholesterol lowering agents, antidepressants). The patients were randomly assigned to exercise or control group. All the patients were taking oral glycemic control medications, no changes in any medications were made throughout the study.

Socio-demographic and clinical data, muscle strength and body mass index were evaluated at the baseline. Fasting blood glucose, hemoglobin A_1C_ (HbA_1C_) and plasma lipid concentration values were measured. Quality of life and symptoms of depression were screened in all patients.

All measurements were done at the same time of the day of evaluation and repeated after eight weeks.

In the exercise group, subjects completed one-repetition maximum (1-RM) strength assessments. Prior to the 1-RM, participants attended one familiarization session. Strength was recorded as the maximal weight lifted and 1 min rest followed by each trial.[14]

Venous blood samples were analyzed for fasting blood glucose, HbA_1C_, total cholesterol, LDL-C, HDL and triglyceride. Sampling was done after a 14-h fast, with no alcohol for the preceding 3 days. Plasma glucose levels were measured by glucose oxidase method.

Metabolic control was determined by HbA_1C_. HbA_1C_ reflects the glycosylization of the hemoglobin molecule, and it is used to assess overall metabolic control over the preceding 8-12 weeks. It is agreed that HbA1C <7% has to be considered as optimal, 7-8% acceptable, and >8% as suboptimal to poor.[15] It was measured by manual colorimetric technique.[16]

Total cholesterol, triglycerides, and HDL-C were determined using commercially available kits from Beckman Coulter. The LDL-C component was estimated using the formula of Friedwald and associates.[17]

Health-related quality of life was evaluated by the Turkish version of Short Form 36. SF-36 includes eight subscales (physical function, physical role, emotional role, social function, bodily pain, mental health, vitality and general health perceptions). A higher SF-36 score indicates better functioning. The Turkish version of the SF-36 has been validated.[18,19]

Centre for Epidemiological Studies-Depression (CES-D) Scale was used to assess depression symptoms. CES-D consists of 20 items that cover affective, psychological, and somatic symptoms. The patient specified the frequency with which the symptom is experienced (that is: a little, some, a good part of the time, or most of the time). It is scored between 0 and 60 points. High scores indicate more severe depressive symptoms.[20]

In the control group, the patients were instructed not to undertake any formal exercise or change their physical activity level during the study period.

Exercise group attended the eight weeks program which consisted of resistance training under the supervision of a physiotherapist, and home- based walking.

Resistance exercises were performed in the exercise room twice in a week. Groups of two or three patients attended these sessions. Each exercise session included approximately 10 min of warm-up and cool down periods. These periods consisted of stretching, and upper- lower body callisthenics. The subjects performed all resistance exercises with 8-10 repetitions in two sets by using free weights. Resistance training intensity commenced at 50% of pretraining 1RM strength tests and increased to 60% in the fourth week of the program. Each resistance exercise took 45-60 s to complete for a total exercise time of approximately 30 min. With the inclusion of pacing for breathing and short rest breaks between some of the exercises, the duration of exercise class was approximately 45 min. The exercises given at each session are listed in Table 1.

**Table 1:** Representative strength and callisthenic exercises

Callisthenic activities	Strength activities
Standing trunk lateral flexion	Standing shoulder flexion with sand bag
Standing with hands behind neck, knee and hip flexion together neck and trunk rotation to same side.	Dumbbell seated biceps curl
Standing ankle circles(with leg extended)	Dumbbell triceps kickback
Standing half squat	Standing hip flexion (ankle weight)
Standing scapular adduction	Standing hip abduction (ankle weight)
Standing shoulder elevation and depression	Sitting knee extension (ankle weight)
Standing shoulder circles to front and back side	Prone knee flexion (ankle weight)
Standing arm swinging	Supine sit ups (no weight)
Sitting on chair neck flexion and lateral flexion	
Sitting on floor, reach to toes	

Walking was performed at a place near home (for example, a garden or a park) without supervision. The subjects were instructed to walk at least twice a week, starting with 15 min per session, and to increase their total weekly walking time by 10 min every two weeks up to 45 min per session. Every walking session included warm-up and cooling down period of 5-10 min. The walking program was designed to be of moderate intensity. At the outset, maximum heart rate was estimated by subtracting one's chronological age from 220.[21] The intensity of training for walk was determined from the individual's maximum heart rate (60< HR_max_ < 79%) which was recommended by the American Diabetes Association (ADA).[22] This protocol was used as the subjects did not undergo exercise stress testing. Pulse palpation method was performed to monitor heart rate. Each patient received an instructional session which included learning to count pulse rate and to monitor heart rate. They were evaluated as to their accuracy in checking their exercise pulse rates. The patients were also examined weekly for monitoring adherence and progress, answering questions and providing individualized feedback related to walking program. This walking program was continued for 8 weeks.

### Statistical analysis

The differences of demographic characteristics and depression scores among the groups were tested with the Mann-Whitney U test. Other measurements were compared before and after the treatment in two groups using analysis of covariance. In analyzing change, main effects for group assignment were examined with the baseline levels of the dependent variables serving as covariates. All statistical analyses were carried out with the Statistical Package for Social Sciences windows version 10, with *p*<0.05 established as the level of significance.

## Results

A total of 38 patients were randomly assigned to exercise or control group at baseline, one of them in each group was withdrawn from the study. They were not included in statistical analyses. No significant adverse events occurred during assessment procedures or the training sessions. Except the BMI, there were no statistically significant differences in demographic and clinical parameters between the two groups at baseline (p>0.05) [Table 2].

**Table 2:** Baseline demographic and clinical parameters in the exercise and control group

Variable	Exercise Group (n = 18)	Control Group (n = 18)	*P* value
Sex (M/F)	15/3	12/6	
Age (year)	51.39 ± 2.02	56.06 ± 1.48	0.14
Duration of diabetes (year)	6.56 ± 1.20	6.86 ± 1.76	0.63
Time of oral hypoglycaemic medication use (year)	4.16 ± 0.82	3.33 ± 1.02	0.13
Anthropometry			
Height (cm)	1.68 ± 0.01	1.67 ± 0.02	0.41
Weight (kg)	80.23 ± 2.26	87.30 ± 4.09	0.29
BMI (kg/m^2^)	28.45 ± 0.95	31.31 ± 1.12	0.03*

Values are means ± SE.

* *P*<0.05

Exercise group's baseline HbA_1C_ was significantly higher than the control groups (p<0.05) [Table 3]; other blood parameters were similar between the two groups (p>0.05) [Table 3].

**Table 3:** Baseline blood parameters in the exercise and control group

Blood chemistries	Exercise group	Control group	Difference	
			
			F value	*P* value
Fasting blood glucose	141.78 ± 7.92	129.11 ± 5.38	2.47	0.12
HbA1C	7.67 ± 0.44	6.75 ± 0.27	5.50	0.02*
Total cholesterol	175.22 ± 5.76	183.83 ± 9.33	1.08	0.30
Triglyce rides	153.33 ± 15.67	162.28 ± 17.64	0.55	0.81
HDL-C	45.48 ± 1.88	51.61 ± 4.65	2.40	0.13
LDL-C	99.06 ± 6.81	99.39 ± 6.29	0.34	0.85

Values are means ± SE.

* *P*<0.05

No significant differences were observed in the depression and four subscales (physical function, physical role, bodily pain, and general health) of the SF-36 between the exercise and control groups (p>0.05). But control groups had significantly higher mental health, emotional role, vitality, social function scores than the exercise group (p<0.05) [Table 4].

**Table 4:** Baseline quality of life and depression scores of the subjects

SF-36	Exercise group	Control group	Difference	
			
			F value	*P* value
Physical function	72.22 ± 14.97	71.66 ± 16.35	0.36	0.55
Physical role	59.72 ± 38.48	72.22 ± 31.95	2.91	0.09
Emotional role	59.25 ± 37.14	83.33 ± 26.1 9	5.93	0.02*
Social function	63.61 ± 16.38	78.89 ± 4.50	6.44	0.01*
Bodily pain	72.05 ± 15.48	73.16 ± 18.41	1.25	0.27
Mental health	55.55 ± 16.22	66 ± 13.24	5.71	0.02*
Vitality	57.5 ± 13.74	63.33 ± 18.39	7.97	0.00*
General health	53.11 ± 20.87	53.11 ± 20.87	0.53	0.46
CES-D#	18.11 ± 7.63	15.61 ± 2.02	-	0.516

Values are means ± SD.

* *P*<0.05

# The groups were compared by Mann–Whitney Test.

Eighteen patients adhered to supervised exercise sessions and home-based walking sessions in the exercise group. Mean walking session was 19.50 ± 4.20. Compliance with the exercise training program, defined as percentage of sessions attended, averaged 96%.

At the end of the study, no significant differences were observed in the body weight, BMI, fasting blood glucose, total cholesterol, LDL-C, HDL-C and triglyceride between the exercise and control groups (p>0.05) [Table 5]. HbA_1C_ was significantly higher in the control group at the end of the study (p<0.05) [Table 5].

**Table 5:** Patients' body weight, BMI and blood values at the end of the study

	Exercise group	Control group	Difference	
			
			F value	*P* value
Body weight	79.67 ± 2.28	87.08 ± 4.06	0.00	0.97
BMI	28.31 ± 0.96	31.24 ± 1.14	0.13	0.72
Fasting blood glucose	11 7.94 ± 6.05	126.33 ± 5.64	1.01	0.32
HbA1C	6.38 ± 0.1 8	6.88 ± 0.29	7.08	0.01*
Total cholesterol	164 ± 5.35	180.55 ± 10.27	2.73	0.10
Triglyce rides	126 ± 10.14	152.61 ± 14.24	1.98	0.16
HDL-C	47.77 ± 2.08	50.72 ± 4.04	1.24	0.27
LDL-C	99.83 ± 5.38	157.22 ± 56.06	1.46	0.23

* *P*<0.05, Values are means ± SE.

In the exercise group, all muscle strengths increased significantly after exercise training [Table 6] (p<0.05).

**Table 6:** Before and after training muscle strengths' values in the exercise group

Variable	Before training	After training	*P* value
Shoulder (kg)	2.83 ± 1.58	3.16 ± 1.57	0.014*
Arm (kg)	4.66 ± 2.16	5.16 ± 2.43	0.003*
Hip flexor (kg)	3.72 ± 1.74	4.16 ± 1.88	0.005*
Hip abductor (kg)	3.05 ± 1.79	3.52 ± 1.89	0.020*
Knee flexor (kg)	3.72 ± 1.74	4.22 ± 1.85	0.008*
Knee extensor (kg)	4.66 ± 2.22	5.22 ± 2.39	0.002*

Values are means ± SD.

* *P*<0.05

Two groups were not different in scores of physical function, physical role, social function, body pain, general health of the SF-36 and depression after study (p>0.05). The exercise group had higher scores of emotional role, vitality and mental health subscales than the control groups after the training programs (p<0.05) [Table 7].

**Table 7:** Patients' SF-36 and depression scores at the end of the study

	Exercise group	Control group	Difference	
			
			F value	*P* value
Physical function	80.83 ± 16.91	73.61 ± 15.97	0.41	0.52
Physical role	90.27 ± 21.24	77.77 ± 29.56	0.619	0.43
Emotional role	88.88 ± 19.80	83.33 ± 26.1 9	4.80	0.03*
Social function	72.22 ± 21.67	81.94 ± 14.98	0.95	0.76
Body pain	80.16 ± 12.48	72.44 ± 18.25	0.55	0.46
Mental health	68 ± 12.49	65.77 ± 13.73	5.96	0.02*
Vitality	68.61 ± 15.51	62.77 ± 19.64	12.12	0.00*
General health	63.38 ± 18.75	53.55 ± 21.66	1.20	0.28
CES-D#	12.88 ± 7.11	14.44 ± 7.50	-	0.48

* P<0.05, Values are means ± SD.

# The groups were compared by Mann–Whitney Test.

## Discussion

Regular exercise offers general as well as diabetes-specific health benefits. Habitual physical activity can reduce risks of coronary heart disease and diabetic complications and enhance quality of life.[23–25] Some studies included only resistance training or aerobic exercise. Findings of these studies shown that patients with diabetes had improved glucose control, increased strength, and decreased cholesterol levels after training.[26–30] The American College of Sports Medicine stated that physical activity, including appropriate endurance and resistance training, is a major therapeutic modality for type-2 diabetes.[7] Following this statement, both resistance and aerobic exercises were studied in diabetic patients.[31,32] These combined studies confirmed exercise-induced improvement in metabolic control and lipid profile. The method of the present study was slightly different from the previous studies that walking which was used to be an aerobic component was practiced as home-based by the patients, whereas resistance training was performed in the outpatient clinic as group-based under the supervision. Our findings are consistent with the previous reports; comparison with the control group indicates that cases in the exercise group have gained more benefits from the treatment. This group showed significant improvements related to glycemic-metabolic control, and quality of life.

We thought that our treatment protocol brought about some advantages. Firstly, group resistance training was carried out in the outpatient clinic by the physiotherapist supervision. Group-based training increased patients' adherence and adaptation to program and efforts during the performing exercises. Majority of the patients said that group approach provided them more willing and enjoyable participation to program. High compliance levels of the patients supported their ideas.

Second part of the program was home-based walking and the patients displayed good adherence to this program. Although we recommended it at least twice a week, sample's mean walking sessions were surprisingly higher than the recommended level. According to literature, enhancement of self efficacy parallel to self management skills resulted in the reduction of patients' glycosylate hemoglobin.[33,34] We believed that walking performed as home program contributed to improvements in either self management skills or metabolic control of the patients. Self care is the cornerstone of diabetes management. Self -monitoring of blood sugar, diet and exercise are important components of self -care activity in diabetes. It was stated that daily consumption of healthy nutritious food and practice of physical activity can slow down disease progression.[35–37] Walking performed by the patients at around their homes might have positively affected self-care and self confidence of the patients; consequently these improvements contributed to improved quality of life in the exercise group.

Increases in LDL and serum triglycerides and decreases in HDL are characteristics for type-2 diabetes patients who have dyslipidemia. Thus exercise could improve lipid profile in diabetic patients.[38,39] In this study, training did not improve lipid profile. In agreement with us, Maiorana *et al*, demonstrated that there were no significant differences in plasma total, HDL or LDL cholesterol, or triglycerides after combined aerobic and resistance training.[32] The more important determinant is the amount of exercise training than the intensity of exercise for plasma lipoproteins.[40] Therefore, it is likely that low training frequency affected inadequate change in lipid profile of our exercise group.

Exercise training is an indispensable component in the medical treatment of patients with type-2 diabetes. Despite the well-known benefits of exercise, unfortunately this modality was ignored by the patients. A goal of this study was to investigate the effects of supervised group and partly home-based exercise program on glycemic and metabolic control, depression, and quality of life in type-2 diabetes patients. According to our observations, such exercise training could be safe, effective, and beneficial in diabetic patients. The training program must be tailored to each diabetic patient's specific limitations, individual needs and possibilities. There is a need for further research, which will be carried out with larger sample groups, to reveal the benefits of different exercise training programs in these patients.
